# Molecular spectrum and differential diagnosis in patients referred with sporadic or autosomal recessive osteogenesis imperfecta

**DOI:** 10.1002/mgg3.257

**Published:** 2016-12-20

**Authors:** Jose A. Caparros‐Martin, Mona S. Aglan, Samia Temtamy, Ghada A. Otaify, Maria Valencia, Julián Nevado, Elena Vallespin, Angela Del Pozo, Carmen Prior de Castro, Lucia Calatrava‐Ferreras, Pilar Gutierrez, Ana M. Bueno, Belen Sagastizabal, Encarna Guillen‐Navarro, Maria Ballesta‐Martinez, Vanesa Gonzalez, Sarenur Y. Basaran, Ruksan Buyukoglan, Bilge Sarikepe, Cecilia Espinoza‐Valdez, Francisco Cammarata‐Scalisi, Victor Martinez‐Glez, Karen E. Heath, Pablo Lapunzina, Victor L. Ruiz‐Perez

**Affiliations:** ^1^ Instituto de Investigaciones BiomédicasConsejo Superior de Investigaciones Científicas‐Universidad Autónoma de MadridMadridSpain; ^2^ CIBER de enfermedades Raras (CIBERER)MadridSpain; ^3^ Human Genetics and Genome Research DivisionCentre of Excellence of Human GeneticsNational Research CentreCairoEgypt; ^4^ Instituto de Genética Médica y Molecular (INGEMM)Hospital Universitario La Paz‐IdiPazUniversidad Autónoma de MadridMadridSpain; ^5^ Orthopedic Surgery Department and Endocrinology DepartmentHospital Universitario de GetafeMadridSpain; ^6^ Unidad de Genética MédicaServicio de PediatríaHospital Universitario Virgen de la ArrixacaMurciaSpain; ^7^ Department of Medical GeneticsFaculty of MedicineIstanbul Medeniyet UniversityIstanbulTurkey; ^8^ Department of GeneticsFaculty of MedicineErciyes UniversityKayseriTurkey; ^9^ Department of GeneticsSchool of MedicinePamukkale UniversityDenizliTurkey; ^10^ Hospital Universitario CatólicoCuencaEcuador; ^11^ Unidad de Genética MédicaDepartamento de PediatríaUniversidad de Los AndesMéridaVenezuela; ^12^ Skeletal Dysplasia Multidisciplinary Unit (UMDE)Hospital Universitario La PazMadridSpain

**Keywords:** Bone development, congenital indifference to pain, Fanconi–Bickel syndrome, osteogenesis imperfecta

## Abstract

**Background:**

Osteogenesis imperfecta (OI) is a heterogeneous bone disorder characterized by recurrent fractures. Although most cases of OI have heterozygous mutations in *COL1A1* or *COL1A2* and show autosomal dominant inheritance, during the last years there has been an explosion in the number of genes responsible for both recessive and dominant forms of this condition. Herein, we have analyzed a cohort of patients with OI, all offspring of unaffected parents, to determine the spectrum of variants accounting for these cases. Twenty patients had nonrelated parents and were sporadic, and 21 were born to consanguineous relationships.

**Methods:**

Mutation analysis was performed using a next‐generation sequencing gene panel, homozygosity mapping, and whole exome sequencing (WES).

**Results:**

Patients offspring of nonconsanguineous parents were mostly identified with *COL1A1* or *COL1A2* heterozygous changes, although there were also a few cases with *IFITM5* and *WNT1* heterozygous mutations. Only one sporadic patient was a compound heterozygote for two recessive mutations. Patients offspring of consanguineous parents showed homozygous changes in a variety of genes including *CRTAP*,*FKBP10*,*LEPRE1*,*PLOD2*,*PPIB*,*SERPINF1*,*TMEM38B*, and *WNT1*. In addition, two patients born to consanguineous parents were found to have de novo *COL1A1* heterozygous mutations demonstrating that causative variants in the collagen I structural genes cannot be overlooked in affected children from consanguineous couples. Further to this, WES analysis in probands lacking mutations in OI genes revealed deleterious variants in *SCN9A*,*NTRK1*, and *SLC2A2*, which are associated with congenital indifference to pain (CIP) and Fanconi–Bickel syndrome (FBS).

**Conclusion:**

This work provides useful information for clinical and genetic diagnosis of OI patients with no positive family history of this disease. Our data also indicate that CIP and FBS are conditions to be considered in the differential diagnosis of OI and suggest a positive role of *SCN9A* and *NTRK1* in bone development.

## Introduction

Osteogenesis imperfecta (OI; MIMs: 166200, 166210, 166220, 166230, 259420, 259440, 610682, 610915, 610967, 610968, 613848, 613849, 613982, 614856, 615066, 615220, 616229, 616507) is a genetically heterogeneous condition characterized by fragile bones which are prone to fracture. This disorder is one of the most frequent skeletal dysplasias with an estimated prevalence of 6‐7/100,000 (Van Dijk et al. 2010). Phenotypically, the severity of OI is variable, extending from affected individuals with mild symptoms to cases with a high number of fractures or even neonatal lethality (Sillence et al. 1979). Additional features commonly found in patients with OI are short stature, bone deformities, wormian bones, dentinogenesis imperfecta, blue sclera, hearing loss, and increased vascular fragility. Typically, OI is associated with defects in the production of collagen type I and consistently 85–90% of OI cases have heterozygous dominant mutations in either *COL1A1* (MIM: 120150) or *COL1A2* (MIM: 120160) encoding procollagen *α*1(I) and *α*2(I) peptide chains, respectively (Chu et al. 1983; Byers and Steiner 1992; Forlino and Marini 2016). Dominant variants in *IFITM5* (MIM: 614757) and *P4HB* (MIM: 176790) have also been described in a few affected individuals, with the latter gene being mutated in the OI‐like disorder named Cole‐Carpenter syndrome (MIM: 112240). *P4HB* codes for the beta subunit of prolyl 4‐hydroxylase, which is implicated in procollagen prolyl hydroxylation and folding, and *IFITM5* is an osteoblast‐specific gene associated with matrix mineralization (Rauch et al. 2015; Forlino and Marini 2016). OI is also transmitted as an autosomal recessive trait in a minority of cases. Patients with autosomal recessive OI (AR‐OI) have been reported with mutations in an increasingly growing collection of genes. Most AR‐OI genes, including *BMP1* (MIM:112264), *CRTAP* (MIM: 605497), *FKBP10* (MIM: 607063), *LEPRE1* (MIM: 610339*)*,* PLOD2* (MIM: 601865), *PPIB* (MIM:123841), *SEC24D* (MIM: 607186)*, SERPINH1* (MIM: 600943), and *TMEM38B* (MIM: 611236) are involved in procollagen (I) post‐translational modifications, processing, folding, secretion, and cross‐linking (Garbes et al. 2015; Forlino and Marini 2016). However, there is also another group of AR‐OI loci, which are not recognized as directly implicated in type I collagen biosynthesis, but play a role in mineralization or osteoblast development. Among this second group of genes are *CREB3L1* (MIM: 616215), *SERPINF1* (MIM:172860), *SP7* (MIM: 606633), *SPARC*, (MIM: 182120), and *WNT1* (MIM:164820) (Mendoza‐Londono et al. 2015; Forlino and Marini 2016). Lastly, mutations in *PLS3* (MIM: 300131) and more recently in *MBTPS2* (MIM: 300294) have been associated with two distinct forms of X‐linked OI (van Dijk et al. 2013; Lindert et al. 2016).

In this work, we have analyzed 42 independent OI probands, all offspring of unaffected parents, to determine in light of the recent advances in the molecular pathogenesis of OI, the spectrum of mutated genes and variants accounting for these cases. In addition, we have identified pathogenic variants in *SCN9A* (MIM: 603415), *NTRK1* (MIM: 191315), and *SLC2A2* (MIM: 138160) in patients with a clinical history compatible with OI.

## Materials and Methods

### NGS sequencing panel

DNA extracted from peripheral blood was subjected to mutation screening using a customized next‐generation sequencing (NGS) gene panel containing 16 genes associated with OI (Appendix S1). This panel was designed with Roche NimbleDesign software (https://design.nimblegen.com). For each sample, paired‐end libraries were created with the help of KAPA HTP Library Preparation Kit for Illumina platforms (Kapa Biosystems, Boston, MA, USA), SeqCap EZ Library SR (Roche Nimblegen, Madison, WI, USA), and NEXTflex‐96 Pre Capture Combo Kit for indexing (Bioo Scientific, Austin, TX, USA). Sequencing was conducted on a MiSeq system (Illumina, San Diego, CA, USA) according to the manufacturer′s standard operating protocol. NGS variants were filtered and those identified as pathogenic were subsequently confirmed by Sanger sequencing and subjected to segregation analysis in the corresponding family.

### SNP arrays

Briefly, 200 ng of genomic DNA from peripheral blood were used to conduct a genome‐wide scan of 850,000 SNPs, using the Illumina CytoSNP‐850k BeadChip following the manufacturer's specifications (Illumina). GenCall scores <0.15 at any locus were considered “no calls”. Hybridization results were analyzed using Genome Studio software (Illumina). Homozygous regions were identified by evaluating allele frequency for all SNPs. Genomic positions were based on NCBI Build 37 (dbSNP version 130).

### Candidate gene screening

For probands of consanguineous families, all coding exons of candidate AR‐OI genes in regions of homozygosity were PCR amplified and sequenced by Sanger sequencing as previously described (Caparros‐Martin et al. 2013). Regarding cDNA analysis, total RNA was obtained from skin primary fibroblasts using Tri Reagent Solution (ThermoFisher Scientific, Waltham, MA, USA). Subsequently, five micrograms of RNA were retrotranscribed with superscript II and random primers (ThermoFisher Scientific) and used for PCR.

### Mutation nomenclature

Mutations are named following the HGVs nomenclature guidelines taking the A of the first ATG as nucleotide +1, and checked with Mutalyzer (http://www.LOVD.nl/mutalyzer) (Wildeman et al. 2008). Variants were considered novel when they were not present in the LOVD OI database maintained by the University of Leicester (https://oi.gene.le.ac.uk) or in HGMD (http://www.hgmd.cf.ac.uk). The following reference sequences were used: *COL1A1*: NM_000088.3; *COL1A2*: NM_000089.3; *CRTAP*: NM_006371.4; *FKBP10*: NM_021939.3; *IFITM5*: NM_001025295.2; *LEPRE1*: NM_022356.3; *PLOD2*: NM_182943.2; *PPIB*: NM_000942.4; *SERPINF1*: NM_002615.5 and NG_028180.1; *TMEM38B*: NM_018112.2 and NG_032971.1; *WNT1*: NM_005430.3; *NTRK1*: NM_002529.3; *SCN9A*: NM_002977.3; *SLC2A2*: NM_000340.1.

### Whole exome sequencing

Whole exome sequencing (WES) was provided by Sistemas Genomicos S.L., Valencia, Spain Briefly, capture and enrichment of DNA targeted regions were performed using the SureSelect Human All Exon Target Enrichment kit for 51 Mb (Agilent Technologies) and exome libraries were sequenced on a HiSeq2000 sequencing platform (Illumina). Reads were aligned against the human reference genome version GRCh37/hg19 and the filtering process was conducted with Picard‐tools (http://picard.sourceforge.net/) and SAM‐tools (Li et al. 2009). A combination of two different algorithms VarScan (Koboldt et al. 2009) and GATK (McKenna et al. 2010) was used for variant calling and identified variants were annotated using the Ensembl database [www.ensembl.org]. Candidate variants were validated by Sanger sequencing.

### In silico analysis of variants

Pathogenicity prediction was obtained using CADD V1.3 (http://cadd.gs.washington.edu) (Kircher et al. 2014), SIFT, Polyphen, and MutationTaster, the last three available in Alamut V2.7 (Interactive Biosoftware, Rouen, France). Splicing alterations were predicted using SplicesiteFinder‐like, MaxEntScan, NNSplice, Genesplicer, and Human Splicing Finder, which are also available in Alamut V2.7 (Interactive Biosoftware).

## Results

### Patients and mutation analysis

We studied 20 sporadic patients offspring of unrelated parents and 22 probands offspring of consanguineous parents, all with clinical diagnosis of OI. For all patients both parents were confirmed to be unaffected. Patients who had nonconsanguineous parents were screened using a customized NGS gene panel containing previously reported OI genes (NGS‐OI). Affected individuals from consanguineous families and their siblings were first subjected to homozygosity mapping using SNP arrays, and subsequently, all the AR‐OI genes contained within candidate homozygous blocks larger than 1 MB were analyzed by Sanger sequencing. For patient 1010, with lethal OI type, both consanguineous parents were tested with the NGS‐OI‐specific gene panel due to lack of proband material. All mutations were verified by Sanger sequencing and confirmed to segregate with the disease in the corresponding family. Missense changes were subjected to in silico pathogenicity analysis and ExAC population allele frequencies were examined for all variants. Appropriate informed consent was obtained from patients or patients’ guardians according to the declaration of Helsinki principles of medical research involving human subjects. This study was approved by the CSIC Institutional Review Board and the Medical Research Ethics Committee of the Egyptian National Research Centre.

### Mutations in sporadic cases offspring of nonconsanguineous parents

We identified heterozygous mutations in *COL1A1* or *COL1A2* in 12/20 sporadic patients of nonrelated parents. In six of them, DNA from both progenitors was available, and the corresponding mutations were confirmed to have arisen as de novo events. Three patients referred with clinical suspect of OI‐V had the recurrent c.‐14C>T *IFITM5* mutation characteristic of this type of OI in the heterozygous state and generated de novo (Cho et al. 2012; Semler et al. 2012), and a 35‐year‐old female described with short stature, vertebral fractures, and early‐onset osteoporosis was found with a heterozygous frameshift mutation in *WNT1*. The same mutation was also detected in the heterozygous state in her mother who only presented osteoporosis after menopause. Finally, one patient was the result of a recessive trait due to compound heterozygous mutations in *SERPINF1* and no mutations were detected in three cases (Table 1). In total, six mutations were novel including two nucleotide deletions in *WNT1* and *SERPINF1* and four glycine substitutions affecting Gly‐XY tripeptide repeats of the *α*1(I) or *α*2(I) chains. Gly‐XY repeats are the main component of *α*‐chains and the requirement of glycines every three amino acids for collagen I triple helix formation has been largely documented (Dalgleish 1997).

**Table 1:** Mutations in sporadic patients offspring of nonconsanguineous parents

Lab family no.	Origin	Gene	Heterozygous mutation	Protein effect	Splicing variant	CADD V1.3	SIFT	Polyphen	Mutation Taster	Population database frequencies	De novo	Novel	OI type/severity score
3	Egypt	*COL1A1*	c.1155+1G>A	p.?	Y					n.p.			I/6
8	Egypt	*COL1A1*	c.1155+1G>C	p.?	Y					n.p.			III/13
12	Egypt	*COL1A1*	c.1299+1G>C	p.?	Y					n.p.			I/10
18	Egypt	*COL1A2*	c.740G>T	p.Gly247Val	–	25.2	Del (0)	Prob dam (1.00)	Disease causing (1.0)	n.p.	Y	Y	III/15
33	Ecuador	*COL1A2*	c.1036G>A	p.Gly346Ser	–	25.9	Del (0)	Prob dam (1.00)	Disease causing (1.0)	n.p.			
39	Spain	*–*											I
46	Spain	*COL1A2*	c.1406G>C	p.Gly469Ala	–	20.3	Del (0)	Prob dam (0.999)	Disease causing (1.0)	n.p.			IV
51	Spain	*COL1A2*	c.1009G>A	pGly337Ser	–	26.5	Del (0)	Prob dam (1.00)	Disease causing (1.0)	n.p.	Y		IV
54	Spain	*COL1A1*	c.3505G>A	p.Gly1169Ser	–	26.4	Del (0)	Prob dam (1.00)	Disease causing (1.0)	n.p.	Y		IV
71	Spain	*–*											IV
72	Spain	*SERPINF1*	Maternal allele: c.271_279dupGCCCTCTCG	p.Ala91_Ser93dup	–					AMR 0.0086%			III
										SAS 0.0061%			
										NFE 0.0045%			
			Paternal allele: c.273_283+1del	p.?	Y					n.p.		Y	
83	Turkey	*COL1A2*	c.1117G>C	p.Gly373Arg	–	32	Del (0)	Prob dam (1.00)	Disease causing (1.0)	n.p.		Y	
88	Turkey	*COL1A1*	c.2533G>A	p.Gly845Arg	–	28.0	Del (0)	Prob dam (1.00)	Disease causing (1.0)	n.p.	Y		
90	Spain	*WNT1*	c.1026delC	p.Glu343Serfs*50	–					n.p.		Y	Early‐onset osteoporosis with fractures
1002	Turkey	*–*											
1005	Spain	*IFITM5*	c.‐14C>T	p.Met1ext‐5	–					n.p.	Y		V
1016	Spain	*IFITM5*	c.‐14C>T	p.Met1ext‐5	–					n.p.	Y		V
1017	Spain	*COL1A1*	c.581G>A	p.Gly194Asp	–	26.1	Del (0)	Prob dam (1.00)	Disease causing (1.0)	n.p.	Y	Y	III
1019	Venezuela	*COL1A2*	c.1073G>A	p.Gly358Asp	–	32.0	Del (0)	Prob dam (1.00)	Disease causing (1.0)	n.p.	Y	Y	III
1027	Spain	*IFITM5*	c.‐14C>T	p.Met1ext‐5	–					n.p.	Y		V

Mutations were classified as novel, when absent from the LOVD database for OI and HGMD. Unknown protein effect: p.?. SIFT: Del: deleterious; Polyphen Prob dam: probably damaging. Allele frequencies were obtained from ExAC with the help of Alamut software. AMR: Hispano‐Americans; NFE: Non‐Finnish Europeans; SAS: South Asians. Variants not present in ExAC are indicated with n.p. OI type (Sillence et al. 1979; Glorieux et al. 2000) and severity score (Aglan et al. 2012) are reported when available. *COL1A1*: NM_000088.3; *COL1A2*: NM_000089.3; *IFITM5*: NM_001025295.2; *SERPINF1*: NM_002615.5; *WNT1*: NM_005430.3.

### Mutations in patients offspring of consanguineous parents

Affected individuals from consanguineous families were identified with homozygous mutations in the following OI recessive genes: *SERPINF1* (four families), *CRTAP* (three families), *FKBP10* (two families), *PLOD2* (two families), *TMEM38B* (two families), *WNT1* (two families), *LEPRE1* (one family), and *PPIB* (one family). No mutations were detected in five cases, and 10 mutations were novel (Table 2). Of the 10 unreported variants, four are predicted to introduce a stop codon either directly or following a frameshift, one was a deep intronic splicing mutation and five corresponded to amino acid substitutions. Missense variants, in addition to not being detected in ExAC, were subjected to in silico analysis which was consistent with pathogenicity in all cases. Only two variants, p.Asp349Gly (c.1046A>G) and p.Met9Val in *CRTAP* and *PPIB*, respectively, showed slightly lower pathogenicity values. Nevertheless, the c.1046A>G variant, located 23 bp upstream of the 3′end of *CRTAP* exon 5, was predicted by five different splice site recognition programs to generate a novel donor splice site (Fig. S1). Likewise, PPIB Met9, which is a conserved residue in vertebrates, has been suggested to be in fact the start codon of *PPIB*, as the protein encoded by this gene was not detected on western blots or by immunofluorescence in fibroblasts from a patient characterized with a Met9Arg homozygous mutation (Barnes et al. 2010). Furthermore, two affected siblings of proband 1011 were found to be homozygous for the p.Met9Val variant, while the sequence of an unaffected brother was normal.

Family 69 was identified with a deep intronic mutation in *SERPINF1*. SNP arrays in the proband of this family and her similarly affected sister were consistent with a mutation in *SERPINF1* as the two siblings shared a 9.1 Mb homozygous region on chromosome 17 encompassing this gene. Since no mutations were detected on sequencing all *SERPINF1* coding exons, we performed RT‐PCR in skin primary fibroblasts using primers covering the entire ORF of the gene. This produced a larger PCR product in the proband with respect to a control, which was found to contain a 63 bp in‐frame insertion between coding exons 5 and 6 by sequencing. Amplification and sequencing of the corresponding genomic region and flanking DNA revealed a homozygous G>A substitution (c.786+715G>A) in the two affected sisters, which creates a novel consensus acceptor splice site (CGG>CAG) that results in the activation of a pseudoexon from intron 5 (Fig. S2). The c.786+715G>A mutation was absent from the 1000 Genome Database.

**Table 2:** Mutations in patients offspring of consanguineous parents

Lab family no.	Origin	Gene	Homozygous mutation	Protein effect	Splicing variant	CADD V1.3	SIFT	Polyphen	Mutation Taster	Population database frequencies	Novel	OI type/severity score	Affected siblings
1	Egypt	*TMEM38B*	g.32476_53457delins ATTAAGGTATA ^Ψ^	p.?	–							III/15	Y
44	Turkey	*TMEM38B*	c.507G>A	p.Trp169*	–					n.p.			Y
53	Egypt	*CRTAP*	c.1046 A>G	p.Asp349Gly	New donor site predicted	21.0	Del (0)	Benign (0.037)	Disease causing (1.0)	n.p.	Y	III/17	No
67	Egypt	*SERPINF1*	c.271_279dupGCCCTCTCG	p.Ala91_Ser93dup	–					AMR 0.0086%		IV/13	No
										SAS 0.0061%			
										NFE 0.0045%			
69	Egypt	*SERPINF1*	c.786+715G>A^Ψ^	p.Lys262_Ile263ins21	Y						Y	IV/12	Y
81	Turkey	*WNT1*	c.421C>T	p.Arg141Cys	–	35.0	Del (0)	Prob dam (1.00)	Disease causing (1.0)	n.p.	Y		No
85	Egypt	*FKBP10*	c.831dupC	p.Gly278Argfs*95	–					AF 0.049%		IV/14	No
										AMR 0.00866%			
										EAS 0.012%			
										NFE 0.015%			
301	Egypt	*SERPINF1*	c.1091 G>A	p.Trp364*	–					SAS 0.0061%	Y	III/13	Y
302	Egypt	*LEPRE1*	c.9dupA	p.Arg4Thrfs*33	–					n.p.	Y	II/lethal	No
89	Egypt	*CRTAP*	c.452T>C	p.Leu151Pro		25.9	Del (0)	Prob dam (0.999)	Disease causing (1.0)	n.p.	Y	III/15	No
91	Egypt	*PLOD2*	c.1358+5G>A	p.?	Y					n.p.		IV/10	No
93	Egypt	*SERPINF1*	c.1091G>A (same as 301)	p.Trp364*	–					SAS 0.0061%		IV/14	No
1004	Egypt	*FKBP10*	c.310C>T	p.Arg104*	–					SAS 0.0061%	Y	BRKS moderate	No
										NFE 0.0045%			
1008	Egypt	*WNT1*	c.506dupG	p.Cys170Leufs*6	–					AFR 0.015%		III/15	No
										SAS 0.028%			
										NFE 0.03%			
1010.m	Sudan	*CRTAP*	c.1112dupT (heterozygous)	p.Tyr372Valfs*2	–					n.p.	Y	II/lethal	Y
1010.f			c.1112dupT (heterozygous)										
1011	Egypt	*PPIB*	c.25A>G	p.Met9Val	–	18.47	Tol (0.13)	Poss dam 0.865	Disease causing (1.0)	n.p.	Y	III/15	Y
1020	Egypt	*PLOD2*	c.1828 T>C	p.Trp610Arg	–	28.5	Del (0)	Prob dam (1.00)	Disease causing (1.0)	n.p.	Y	III/17 BRKS severe	Y

Mutations were classified as novel, when absent from the LOVD database for OI and HGMD. Only the parents of case 1010 were tested owing to neonatal lethality. The same mutation was identified in the heterozygous state in the two progenitors. Unknown protein effect: p.?. SIFT: Del: deleterious; Tol: tolerated; Polyphen Prob dam: probably damaging; Poss dam: possibly damaging. Allele frequencies were obtained from ExAC with the help of Alamut software. AFR: Africans; AMR: Hispano‐Americans; EAS: East Asians; NFE: Non‐Finnish Europeans; SAS: South Asians. Variants not present in ExAC are indicated as n.p.. Sillence OI type (Sillence et al. 1979) and severity score (Aglan et al. 2012) are reported when available. Mutations indicated with Ψ are explained in Appendix S1. *CRTAP*: NM_006371.4; *FKBP10*: NM_021939.3; *LEPRE1*: NM_022356.3; *PLOD2*: NM_182943.2; *PPIB*: NM_000942.4; *SERPINF1*: NM_002615.5 and NG_028180.1; *TMEM38B*: NM_018112.2 and NG_032971.1; *WNT1*: NM_005430.3.

### Disparity of phenotypes associated with *PLOD2* mutations

Egyptian families 91 and 1020 were linked to *PLOD2* by homozygosity mapping. This gene was contained within a 32 Mb homozygous block in the index patient of family 91 and within a region of homozygosity of 90 Mb shared by the two affected siblings of family 1020. Sequencing of *PLOD2* in the proband of each family revealed a homozygous donor splice site mutation in intron 12 (c.1358+5G>A) in the affected girl of family 91, previously reported by our group in another Egyptian patient (Puig‐Hervas et al. 2012), and a novel homozygous missense mutation, p.Trp610Arg, in the affected sisters of family 1020. The new amino acid change involves a highly conserved residue from exon 16 which is adjacent to the exon 17 cluster of missense mutations (R619H, G622V, G622C, V627A, T629I) associated with Bruck syndrome 2 (BRKS2; MIM: 601865) (van der Slot et al. 2003; Ha‐Vinh et al. 2004; Puig‐Hervas et al. 2012; Zhou et al. 2014). BRKS is a recessively inherited form of OI that includes congenital contractures as a distinguishing feature (McPherson and Clemens 1997) and is caused by *PLOD2* or *FKBP10* mutations (Shaheen et al. 2010). Notably, the patients from families 91 and 1020 showed quite different phenotypes (Fig. S3). The proband from family 91 is a 9‐year‐old girl with no congenital contractures. Her first fracture occurred at 6 years of age and afterward there was an average of 1–2 fractures/year. She had a short stature (height: −4.0 SD; weight: +0.4 SD; head circumference: +0.3 SD), blue sclera, very mild hypotonia, wormian bones, and osteoporosis revealed by DEXA (total body Z‐score: −2.5 and forearm *Z*‐score: −4.4). In contrast, the two sisters from family 1020 had a very severe form of BRKS. Both, the proband (13 years and 7 months old) and her younger sister (4 years and 8 months old) presented grayish blue sclera, congenital joint contractures, bone deformities, kyphoscoliosis, and muscle wasting of lower limbs. They were both underweight (−4.3 and −3.3 SD, respectively), short (−12.3 and −5.0 SD, respectively), and microcephalic (−6.0 and −4.0 SD, respectively). Fractures started 3 days after birth in the proband and at birth in the other sibling and recurred with an average of 10 fractures per year with severe bone pain. X‐ray of upper and lower limbs in both sisters revealed thin bowed serpentine long bones with metaphyseal widening, honey comb appearance, and multiple fracture sites with callus formation. DEXA for the proband demonstrated severe osteoporosis (spine *Z*‐score: −5.5 SD). In addition to craniosynostosis and severe developmental delay (SQ: 26 by Vineland Social Maturity Scale), the younger sister had facial palsy since birth. 3D skull scan CT revealed bilateral premature closure of coronal and lambdoid sutures, closed posterior fontanelle, preserved anterior fontanelle, prominent cortical sulci, basal cistern, and dilatation of ventricular system.

### Differential diagnosis of OI

Five of the 22 probands of consanguineous families yielded a negative result for mutations in AR‐OI genes. These patients were either not linked to any of the reported AR‐OI loci by homozygosity mapping or were detected with no mutations after sequencing the candidate genes embedded in the corresponding homozygous regions. As this suggested the possibility of additional OI genes, we performed WES in four of these patients (families 16, 30, 84, 1007). The fifth proband was analyzed with the NGS‐OI panel (family 1009). Despite parental consanguinity, the two affected children from families 30 and 1009 were found to have *COL1A1* heterozygous mutations which were demonstrated to be de novo following analysis of the corresponding sequence of their progenitors (Table 3). WES data in the three other remaining probands showed no pathogenic changes in *COL1A1/2* or any other OI genes, and consequently, this prompted us to search for rare variants contained within the candidate homozygous regions previously uncovered by the SNP arrays. As a result, we identified a homozygous nonsense mutation (p.Trp190*) in the voltage‐gated sodium channel encoded by *SCN9A* in the proband of family 84 that was also in homozygosis in her affected aunt, and a homozygous missense change involving a conserved residue of the extracellular domain of the neurotrophic receptor tyrosine kinase 1, *NTRK1* (p.Pro311Leu) in the index patient of family 16 (Fig. S4). Remarkably, *SCN9A* is associated with congenital indifference to pain (CIP; MIM: 243000) and *NTRK1* with congenital insensitivity to pain with anhidrosis (CIPA, MIM: 256800) (Mardy et al. 1999; Cox et al. 2006). The third proband, from family 1007, had a missense mutation in *SLC2A2* (p.Gly119Arg), affecting also a conserved amino acid. *SLC2A2* codes for a glucose transporter and is mutated in Fanconi–Bickel syndrome (FBS; MIM: 227810) (Santer et al. 1997). The selected variants in *SCN9A*,* NTRK1*, and *SLC2A2* were not in the ExAC database, were confirmed to segregate with the disease in the three families and were estimated as pathogenic by the four in silico programs we used for predicting pathogenicity (Table 3). To the best of our knowledge, they represent novel mutations. Subsequent re‐evaluation of the phenotype was consistent with the molecular findings especially in patients from families 84 and 1007 (Appendix S1). The proband from family 16 had dry skin and anhidrosis as occurs with CIPA patients having *NTRK1* mutations, but could still feel pain and superficial sensations, which suggests that the p.Pro311Leu substitution is likely to act as a hypomorphic mutation (Appendix S1for clinical descriptions and Fig. S4).

**Table 3:** Mutations in patients offspring of consanguineous parents with no mutations in AR‐OI genes

Lab family no.	Origin	Gene	Mutation	Protein effect	Splicing variant	CADD V1.3	SIFT	Polyphen	Mutation taster	Population database frequencies	Novel	OI type/severity score	Affected siblings
16	Egypt	*NTRK1*	c.932C>T (homozygous)	p.Pro311Leu	–	33.0	Del (0)	Prob dam (1.00)	Disease causing (1.0)	n.p.	Y	IV/11	No
30	Egypt	*COL1A1*	c.3226G>A (de novo; heterozygous)	p.Gly1076Ser	–	25.3	Del (0)	Prob dam (1.00)	Disease causing (1.0)	n.p.		III/15	No
84	Egypt	*SCN9A*	c.570G>A (homozygous)	p.Trp190*	–					n.p.	Y	IV/9	Affected aunt
1007	Egypt	*SLC2A2*	c.355G>A (homozygous)	p.Gly119Arg	–	27.6	Del (0)	Prob dam (0.983)	Disease causing (1.0)	n.p.	Y	FBS	No
1009	Egypt	*COL1A1*	c.2299G>A (de novo; heterozygous)	p.Gly767Ser	–	34	Del (0)	Prob dam (0.999)	Disease causing (1.0)	n.p.		III/13	No

Mutations were classified as novel when absent from LOVD database for OI or HGMD. SIFT: Del: deleterious; Polyphen Prob dam: probably damaging. The five variants were not present in ExAC (n.p.). Sillence OI type (Sillence et al. 1979) and severity score (Aglan et al. 2012) are indicated. *COL1A1*: NM_000088.3; *COL1A2*: NM_000089.3; *NTRK1*: NM_002529.3; *SCN9A*: NM_002977.3; *SLC2A2*: NM_000340.1.

## Discussion

Herein, we have characterized the mutation spectrum of a large cohort of OI patients with no parental positive history of this disease. The results of this study show that sporadic patients from nonconsanguineous relationships commonly have heterozygous *COL1A1* or *COL1A2* mutations generated de novo, whereas mutations in other dominant genes or compound heterozygous mutations in recessive genes are rare in these cases. Sporadic patients with OI type V represent an exception, since they consistently have the c.‐14C>T *IFITM5* mutation typical of this type of OI (Rauch et al. 2013). In a patient with a mild phenotype, initially thought as sporadic, we found a new dominant pathogenic variant in *WNT1*. Interestingly, the mother of this patient carried the same mutation and her only complaint was osteoporosis after menopause. Thus, *WNT1* mutant variants not only can result in dominant or recessive inheritance [(Keupp et al. 2013) and Tables 1 and 2], but can also show variable expressivity. No mutations were detected in three sporadic cases born to nonrelated parents following NGS‐OI panel testing. Although this could be an indication of additional unraveled causative OI genes for these cases, it needs to be taken into account that deep intronic mutations or dosage variations have not been addressed by our screening methodology. Further studies in these three patients including WES analysis in a trio manner (proband and parents) are required to clarify the underlying genetic defect in each of these patients. In consanguineous families, all the affected subjects were identified with homozygous changes in recessive genes except for two probands who to our surprise had de novo *COL1A1* pathogenic variants. Thus, screening of *COL1A1* and *COL1A2* is recommended also in consanguineous families. Concerning AR‐OI genes, our study has confirmed in additional patients, that mutations in *PLOD2* are associated with phenotypes of variable severity ranging from mild OI to very severe BRKS. This has previously been reported only once, most likely due to the low rate of *PLOD2* mutations (Puig‐Hervas et al. 2012). The *PLOD2* homozygous change (c.1358+5G>A) detected in the proband of family 91 was previously reported by our group in another independent Egyptian patient of consanguineous parents (Puig‐Hervas et al. 2012). In this report, the c.1358+5G>A variant was subjected to minigene analysis and in this system, no production of wild‐type product was observed, indicating that it is likely to be a fully penetrant mutation (Puig‐Hervas et al. 2012). Comparing the phenotype of the two patients with the same *PLOD2* mutation, both have no contractures and white sclera, but differ in the severity of the condition. The patient reported in Puig‐Hervas et al. 2012 had more than 10 fractures per year and kyphoscoliosis with a clinical severity score (CSS) of 16 (severe) (Aglan et al. 2012). In contrast, the patient of this study presented 1–2 fractures per year and mild kyphosis, with a CSS of 10 (moderate severity). Further to this, the proband of family 91 was identified with myopathy evidenced by electromyogram and muscle biopsy histochemistry also revealed selective type 2 atrophy as a nonspecific myopathic feature. This finding was not observed in the patient reported earlier with the same mutation. Because exome sequencing was not performed in proband 91, the myopathy of this patient could result from another variant in a different gene. However, it should be noted that abnormalities in muscle fibers in addition to bone fragility have been described in a recently developed *plod2* mutant zebrafish, and thus there is also the possibility of the muscle dysfunction of this patient being part of the phenotypic spectrum associated with mutations in *PLOD2* (Gistelinck et al. 2016).

In family 1020, one of the affected sisters also identified with a *PLOD2* mutation, had in addition to bone fragility and congenital contractures, an unusual association of craniosynostosis and microcephaly with intellectual disability. Since this is not a typical feature of BRKS and because of the parental consanguinity, we are inclined to think that this is likely to be due to a different associated condition for which we cannot figure out a definite syndrome.

Our data also suggest that mutations in *SERPINF1* might be the most frequent finding among families with recessive inheritance. In agreement with this, while this manuscript was under review, a study of 598 OI individuals was published in which *SERPINF1* followed by *CRTAP* were also identified as the genes more frequently mutated in AR‐OI (Bardai et al. 2016). Nevertheless, this has to be confirmed by further studies.

Three patients from two independent consanguineous families with clinical features suggestive of OI proved to have mutations in *SCN9A* (two patients) and *NRTK1* (one patient) which are associated with CIP, and an additional patient had a missense change in the FBS gene *SLC2A2*. Hence, these two conditions should be considered in the differential diagnosis of OI, especially during the first years of life when other distinctive clinical signs of CIP or FBS are not evident. CIP patients are often reported with recurrent bone fractures (Schulman et al. 2001; Perez‐Lopez et al. 2015; Phatarakijnirund et al. 2016), and although this could be explained as a consequence of the absence of pain perception, it could also be indicative of a possible role of the nociceptive neurons in bone homeostasis. The *NTRK1* patient from this study is pointing to the latest as this girl was found to have osteoporosis by DEXA (Appendix S1); however, further DEXA studies of CIP patients are required to be able to draw a reliable conclusion. How nociceptive fibers could influence the biology of bone is largely unknown, although there exists the possibility that they could regulate bone remodeling. Relative to this, blockage of Cav2.2 channels in nociceptors was shown to induce the osteoclast activator RANKL in a preclinical joint inflammation model generated in mice (Baddack et al. 2015). The connection between *SLC2A2* and bone fragility is much clear since hypophosphatemia and rickets are key clinical features of FBS and low phosphate levels result in deficient bone mineralization. The inactivation of the SLC2A2 glucose transporter impairs renal tubular reabsorption of phosphate due to proximal renal tubular dysfunction secondary to glycogen accumulation. Differential diagnosis of OI has been previously hinted in FBS patients by Hadipour et al. 2013, who reported a 3.5 year girl presenting with recurrent bone fractures and atypical clinical presentation that was initially diagnosed as OI, but whose correct diagnosis as FBS was confirmed by molecular studies (Hadipour et al. 2013). There are also other reports indicating that wide phenotypic variability adds difficulty for accurate diagnosis of such rare disorder. For example, atypical or mild presentation with absent hepatomegaly and short stature in two sisters was described by Grunert et al. 2012. Our patient at presentation had features of nutritional rickets which improved with treatment. On follow up, he showed short stature, growth retardation, mild hepatomegaly, fasting hypoglycemia but no glucosuria nor aminoaciduria. He also had multiple fractures, wormian bones, and severe osteoporosis in DEXA, which are typical characteristics of OI, thus making it difficult to reach a definite diagnosis by clinical assessment only. Similarly, the patient with *NTRK1* mutation had on one hand repeated fractures and osteoporotic features, and on the other anhidrosis, but could feel pain and superficial sensations. Our results emphasize the importance of molecular data to confirm the diagnosis and help in proper counseling and management. Particularly, in developing countries with limited access to advanced molecular studies, close and regular follow‐up of patients with recurrent fractures is of utmost importance to check for other emerging symptoms and signs that appear with time and can change the diagnosis.

## Supporting information


**Appendix S1.** Clinical description of patients with mutations in CIP genes and *SLC2A2*.
**Figure S1.** *CRTAP* c.1046A>G (p.Asp349Gly) may create a new donor splice site.
**Figure S2.** Mutations in consanguineous families 1 and 69.
**Figure S3.** Clinical images of patients from families 91 (A) and 1020 (B) with mutations in *PLOD2*, demonstrating differences in phenotype severity and the presence of joint contractures only in the affected sisters of family 1020.
**Figure S4.** *SCN9A*,* NTRK1*, and *SLC2A2* mutations in patients with OI skeletal features.
