# Supplementary material for: Molecular spectrum and differential diagnosis in patients referred with sporadic or autosomal recessive osteogenesis imperfecta
Source: Mol Genet Genomic Med. 2016 Dec 20;5(1):28–39. doi: 10.1002/mgg3.257 (PMC5241205; doi:10.1002/mgg3.257)
Supplement: Supplementary file 1 — Appendix S1. Clinical description of patients with mutations in CIP genes and SLC2A2. Figure S1. CRTAP c.1046A>G (p.Asp349Gly) may create a new donor splice site. Figure S2. Mutations in consanguineous families 1 and 69. Figure S3. Clinical images of patients from families 91 (A) and 1020 (B) with mutations in PLOD2, demonstrating differences in phenotype severity and the presence of joint contractures only in the affected sisters of family 1020. Figure S4. SCN9A, NTRK1, and SLC2A2 mutations in patients with OI skeletal features. [file MGG3-5-28-s001.pdf]

SUPPLEMENTAL INFORMATION

CLINICAL DESCRIPTION OF PATIENTS WITH MUTATIONS IN CIP GENES AND *SLC2A2*

Family 84. The proband of family 84 presented to us at age 6 months. She was a female born to double first cousin parents who had an unaffected sister. She had three fractures at 3 months of age, and grayish sclera with similarly affected aunt that has history of multiple fractures, so she was suspected to have OI. On further evaluation the parents reported another fracture at 13 months of age followed by cyanosis and sudden abdominal distension requiring hospitalization and died after 2 weeks at 14 months of age. On re-evaluation she was found to have a behavior consistent with insensitivity to pain. By asking the mother about pain sensation in the affected daughter, she reported insensitivity to pain during injections and taking blood samples during her hospital stay. Her aunt, also a product of consanguineous parents, is a 14 years old female. She had four fractures in both arms in the first year of life with subsequent deformities. Lower limbs (LL) also started to develop bowing at age of 6-7 months old without fractures. Additionally, she developed a knee abscess that was followed by unequal length of LL. Insensitivity to pain was also reported, but she refused to come to us for proper assessment of pain and smell senses.

Family 16. The proband of family 16 is a nine year old female patient, offspring of first cousin parents. She had two unaffected sisters that were proved not to be homozygous for the *NTRK1* p.Pro311Leu mutation identified in the proband. Hyperextensibility of joints was noted since birth, while spontaneous fractures of right leg were first observed

at the age of 7 years. Two previous old malunited fractures at ankles were detected by radiological examination. She had blue sclera, asymmetry of gluteal regions, hyperextensibility of interphalangeal joints, wrists and ankles, bilateral clinodactyly of 5th fingers, medial deviation of left knee, edematous ankle joints and dry skin and anhidrosis. Tone and reflexes were normal. Radiological examination revealed dislocation of left femur with irregularities and decreased bone density of femoral head, evidence of multiple fractures at the lower end of femur, lower one third of left fibula and lower part of left tibia in addition to excessive pneumatization of petrous skull bone. Magnetic resonance imaging (MRI) of both ankles showed an old neglected non-united fracture at the super-lateral aspect of the left talus with avascular necrosis on top and chronic low grade synovitis and synovial effusion of both ankle joints causing bilateral ankle edema. MRI of lumbar spine was normal. Anthropometric measurements including height, weight and head circumference were consistent with age. Hearing test was normal. Electromyogram (EMG) and nerve conduction studies were normal. Calcium, phosphorous and alkaline phosphatase levels were within normal limits. Bone densitometry (DEXA) revealed osteoporosis of lumbar spine, left proximal femur, right greater trochanter and left distal radius with moderate osteopenia of right femoral head. The patient has anhidrosis, but can feel pain and superficial sensations and there was no self-mutilation.

Family 1007. The proband of family 1007 is a three years and 6 months old male, born to first cousin parents, with no additional siblings. His first complaint was delayed motor milestones and delayed closure of anterior fontanel. EMG and MRI for the brain were normal but he presented mild mental retardation (IQ: 66). Since levels of calcium and phosphorous were low and alkaline phosphatase high in addition to rachitic

manifestations in X-rays, he was first managed as calcipenic rickets that improved on Ca and vitamin D supplements, which led us to think that he had nutritional rickets being this common in our community. Because hypotonia and delayed motor skills persisted, he received physiotherapy during which he developed his first fracture at 2.5 years and second fracture at age 3 years. DEXA revealed severe osteoporosis and skull X-ray showed wormian bones. He also had greyish sclera. Those three findings in addition to repeated fractures led us to suspect OI. On re-assessment he was found to have clinical signs consistent with FBS including polyuria, polydipsia, occasional facial oedema which may be an indication of nephropathy, and abdominal enlargement. All these features were not found at his initial examination. Laboratory investigations showed fasting hypoglycemia, but normal post-prandial glucose. Urine analysis showed mild proteinuria but no aminoaciduria, glucosuria nor phosphaturia. Abdominal US revealed mild hepatomegaly.

OI-NGS PANEL

All coding exons of the following genes were included in the NGS-OI screening panel: *BMP1*, *COL1A1*, *COL1A2*, *CREB3L1*, *CRTAP*, *FKBP10*, *IFITM5*, *LEPRE1*, *PLOD2*, *PLS3*, *PPIB*, *SERPINF1*, *SERPINH1*, *SP7*, *TMEM38B*, *WNT1*.

PIPELINE USED IN NGS-WES DATA

NGS-WES variants were analyzed with the following filter: Minimum coverage 10 reads (DP≥10); Rate of altered allele >0.75 (alt_rate>0.75); Minor allele frequency in 1000 genomes < 0.001 (MAF_1000G < 0.001); Not in dbSNP; Eliminated variants

annotated as downstream/upstream/intergenic/within non coding genes. The summary of identified variants is in the following table:

Proband	16	84	1007
Total number of variants after filtering	99	26	17
Variants in candidate regions of homozygosity	2	7	7
Predicted as pathogenic by more than one prediction program (Shift +Polyphen)	1	3 (only one present in homozygosis in the affected aunt)	1
Gene;<br>Variant position on human GRCh37/hg19 assembly;<br>Size of the homozygous block containing the variant;<br>Protein effect	*NTRK1*<br>*chr1:156843506C>T*<br>44.5 Mb<br>(p.Pro311Leu)	*SCN9A*<br>*chr2:167162328C>T*<br>24.3 Mb<br>(p.Trp190*)	*SLC2A2*<br>*chr3:170732274C>T*<br>45 Mb<br>(p.Gly119Arg)

SUPPLEMENTARY FIGURE 1

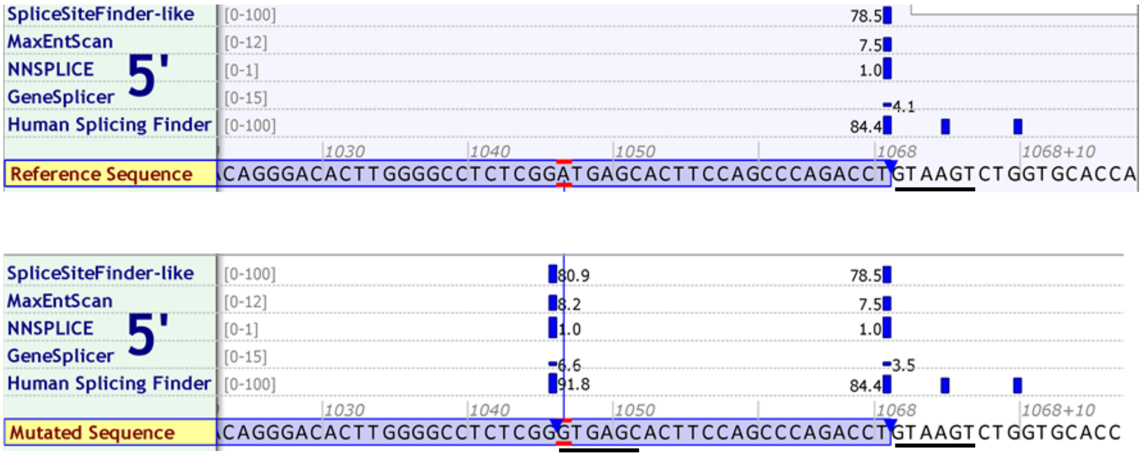

Supplementary figure 1. *CRTAP* c.1046A>G (p.Asp349Gly) may create a new donor splice site. Image from Alamut software (Interactive Biosoftware) showing normal (upper panel) and mutant (lower panel) sequence from *CRTAP* exon 5. Exon 5 sequence is boxed, and the position of the nucleotide mutation is indicated with red lines. Five different splice-site recognition programs predict the creation of a new donor splice site by the c.1045A>G mutation which is scored higher than the normal donor site of this exon. Donor splice sites are underlined.

SUPPLEMENTARY FIGURE 2

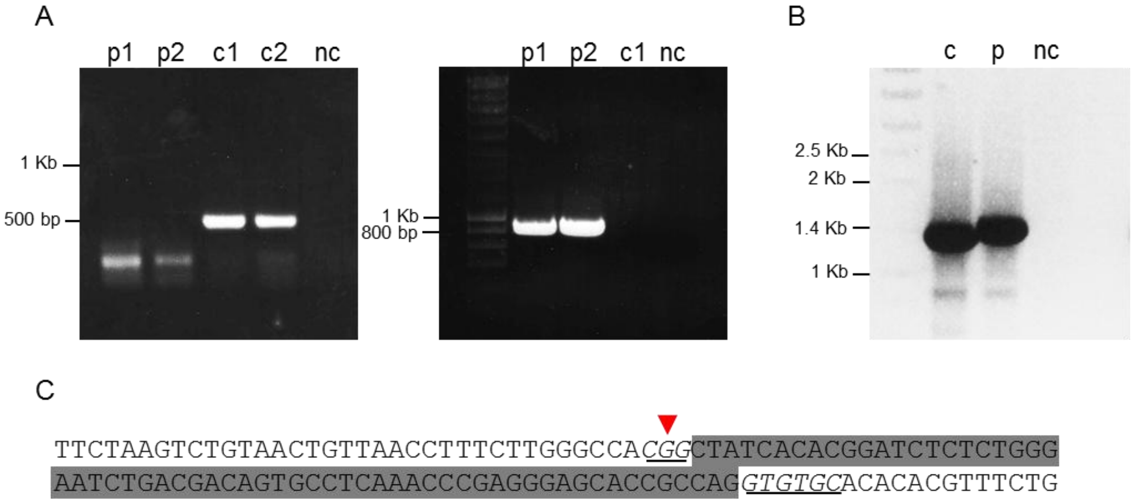

Supplementary figure 2. Mutations in consanguineous families 1 and 69. A. Patients of family 1 are homozygous for the recurrent *TMEM38B* exon 4 deletion reported in Bedouin families (Shaheen, et al., 2012; Volodarsky, et al., 2013). Left side: PCR analysis of genomic DNA using primers contained within the reported deleted fragment showing failure to amplify the corresponding band (503 bp) in the two affected brothers of family 1 (p1, p2), but not in control individuals (c1, c2). The smaller PCR bands of faint appearance in p1 and p2 are non-specific products. Right side: PCR amplification of genomic DNA using primers reported to flank the Bedouin exon 4 deletion showing amplification of the expected 821 bp fragment only in patients (Volodarsky, et al., 2013). Sequencing of the 821 bp PCR product confirmed the NG_032971.1:g.32476_53457delinsATTAAGGTATA recurrent mutation. B. RT-PCR from skin primary fibroblasts with primers covering the entire *SERPINF1* coding region. The RT-PCR product of the proband of family 69 (p) is slightly larger than the product amplified in cDNA from a control individual (c). Lanes named nc in A-B are no template controls. C. Nucleotide sequence corresponding to the 63 bp cryptic exon (grey box) incorporated into the *SERPINF1* cDNA in the patients from family 69. The NG_028180.1: c.786+715G>A substitution (red arrowhead) generates a canonical 3´-

acceptor splice site (CGG>CAG). The 3´ and 5´ splice sites of the cryptic exon are underlined.

SUPPLEMENTARY FIGURE 3

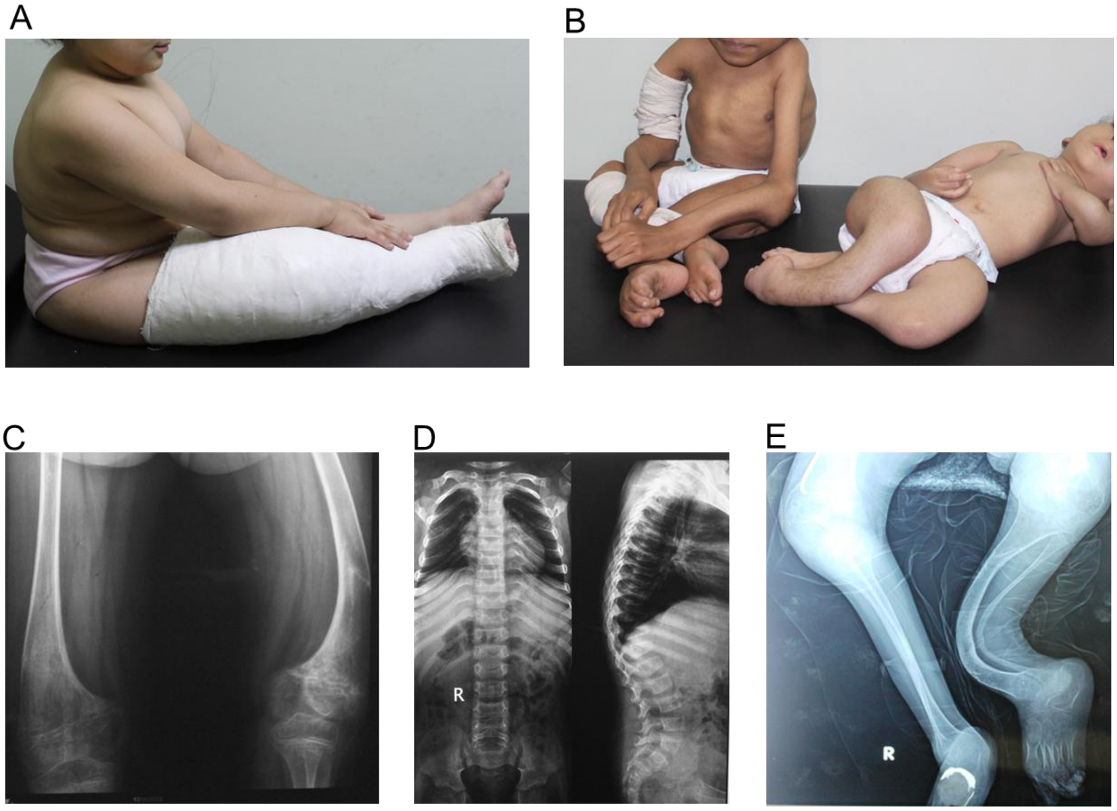

Supplementary figure 3. Clinical images of patients from families 91 (A) and 1020 (B) with mutations in *PLOD2*, demonstrating differences in phenotype severity and the presence of joint contractures only in the affected sisters of family 1020. A. Patient from family 91 (c.1358+5G>A) with cast on right LL due to fracture of femur, no congenital joint contractures and mild bone deformities. B. Affected sibs from family 1020 (p.Trp610Arg) showing severe bone deformities in the form of pectus carinatum, sabre tibiae and bowing of long bones in addition to congenital joint contractures. C-D. X-rays of the proband of family 91. C. Anteroposterior (AP) view of LL showing bowing of left femur, metaphyseal widening of lower ends of femora and honey comb appearance. D. AP and lateral views of spine showing mild kyphosis, platyspondyly of

lower thoracic vertebrae and irregular ribs. E. X-Ray of LL (AP view) of the proband of family 1020 showing serpentine long bones, metaphyseal widening and rod inserted in right bowed femur.

SUPPLEMENTARY FIGURE 4

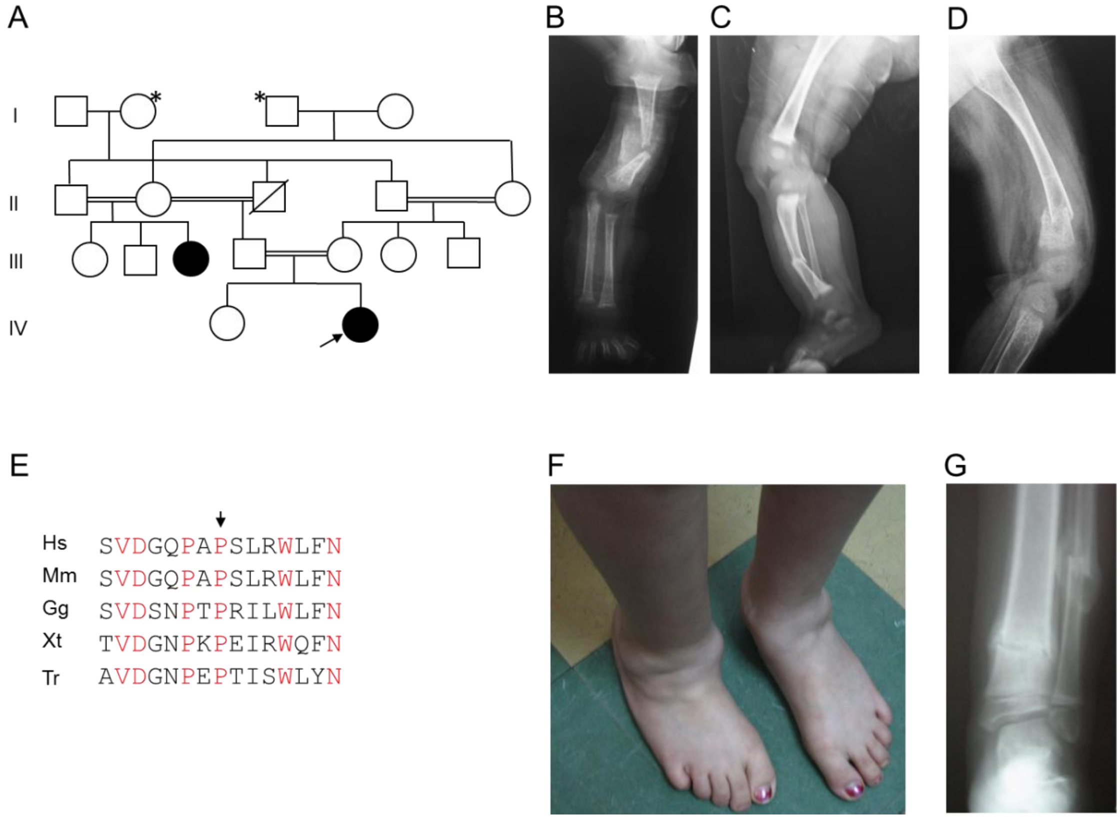

Supplementary figure 4. *SCN9A*, *NTRK1* and *SLC2A2* mutations in patients with OI skeletal features. A. Pedigree structure of family 84. Asterisks in individuals I-II and I-III denote that they were related (second cousins). Affected individuals IV-2 and III-3 were both homozygous for the *SCN9A* c.570G>A nonsense mutation, III-2, III-4 and III-5 had the mutation in the heterozygous state and IV-1 showed normal sequence. DNA from the remaining pedigree members was not available. B-C. Skeletal X-ray showing fractures in humerus, and right tibia of the proband from family 84. D. X-ray left LL (lateral view) of the proband of family 1007 showing fracture at the lower third

of femur in addition to rachitic manifestations in the form of flaring of distal femoral metaphysis. E. Amino acid alignment of a NTRK1 fragment demonstrating conservation of Proline 311 (arrow) in different vertebrates. Invariable residues are shown in red. Hs (*Homo sapiens*), Mm (*Mus musculus*), Gg (*Gallus gallus*), Xt (*Xenopus tropicalis*), Tr (*Takifugu rubripes*). F. Bilateral ankle edema due to synovitis in the patient from family 16 with *NTRK1* mutations. G. X-ray of lower left leg of the patient from family 16 showing fractures at lower end of tibia and lower third of fibula with periosteal reaction.

SUPPLEMENTARY REFERENCES

Shaheen R, Alazami AM, Alshammari MJ, Faqeih E, Alhashmi N, Mousa N, Alsinani A, Ansari S, Alzahrani F, Al-Owain M and others. 2012. Study of autosomal recessive osteogenesis imperfecta in Arabia reveals a novel locus defined by TMEM38B mutation. J Med Genet 49(10):630-5.

Volodarsky M, Markus B, Cohen I, Staretz-Chacham O, Flusser H, Landau D, Shelef I, Langer Y, Birk OS. 2013. A deletion mutation in TMEM38B associated with autosomal recessive osteogenesis imperfecta. Hum Mutat 34(4):582-6.
